# Implementing the communication for development strategy to improve knowledge and coverage of measles vaccination in western Chinese immunization programs: a before-and-after evaluation

**DOI:** 10.1186/s40249-017-0261-y

**Published:** 2017-04-24

**Authors:** Ming Lu, Yao-Zhu Chu, Wen-Zhou Yu, Robert Scherpbier, Yu-Qing Zhou, Xu Zhu, Qi-Ru Su, Meng-Juan Duan, Xuan Zhang, Fu-Qiang Cui, Hua-Qing Wang, Yi-Biao Zhou, Qing-Wu Jiang

**Affiliations:** 1 School of Public Health, Fudan University, Shanghai, People’s Republic of China; 2 National Immunization Program, Chinese Center for Disease Control and Prevention, Beijing, People’s Republic of China; 3 UNICEF Beijing Office, Beijing, People’s Republic of China

**Keywords:** Communication for development, Immunization knowledge, Measles vaccine coverage, Evaluation

## Abstract

**Background:**

Communication for Development (C4D) is a strategy promoted by the United Nations Children’s Fund to foster positive and measurable changes at the individual, family, community, social, and policy levels of society. In western China, C4D activities have previously been conducted as part of province-level immunization programs. In this study, we evaluated the association of C4D with changes in parental knowledge of immunization services, measles disease, and measles vaccine, and changes in their children’s measles vaccine coverage.

**Methods:**

From April 2013 to April 2014, C4D activities were implemented as part of provincial immunization programs in the Inner Mongolia, Guangxi, Chongqing, Guizhou, Tibet, Shaanxi, Gansu, Ningxia, and Qinghai provinces. We used a before-and-after study design and employed face-to-face interviews to assess changes in parental knowledge and vaccination coverage.

**Results:**

We surveyed 2 107 households at baseline and 2 070 households after 1 year of C4D activities. Following C4D, 95% of caregivers were aware of the vaccination record check requirement for entry into kindergarten and primary school; 80% of caregivers were aware that migrant children were eligible for free vaccination; more than 70% of caregivers knew that measles is a respiratory infectious disease; and 90% of caregivers knew the symptoms of measles. Caregivers’ willingness to take their children to the clinic for vaccination increased from 51.3% at baseline to 67.4% in the post-C4D survey. Coverage of one-dose measles-containing vaccine (MCV) increased from 83.8% at baseline to 90.1% after C4D. One-dose MCV coverage was greater than 95% in the Guangxi, Shaanxi, and Gansu provinces. Two-dose MCV coverage increased from 68.5 to 77.6%. House-to-house communication was the most popular C4D activity among caregivers (91.6% favoring), followed by posters and educational talks (64.8 and 49.9% favoring).

**Conclusions:**

C4D is associated with increased caregiver knowledge about measles, increased willingness to seek immunization services for their children, and increased measles vaccination coverage. Tailored communication strategies based on insights gained from these analyses may be able to increase vaccination coverage in hard-to-reach areas. C4D should be considered for larger scale implementation in China.

**Electronic supplementary material:**

The online version of this article (doi:10.1186/s40249-017-0261-y) contains supplementary material, which is available to authorized users.

## Multilingual abstracts

Please see Additional file 1 for translations of the abstract into five official working languages of the United Nations.

## Background

Infectious diseases are associated with poverty [1, 2]. Reducing the burden of infectious diseases in developing countries is important, as recognized in the United Nations Millennium Development Goals [3–6]. An increasing number of diseases are being controlled or even eliminated with vaccines. However, controlling or eliminating childhood diseases with vaccines depends on caregiver compliance with the recommended immunizations [7–9]. China wanted to eliminate measles in 2012, a goal that requires attaining and sustaining a uniformly high coverage of measles vaccine.

In recent decades, the Chinese government’s implementation of the Expanded Program on Immunization (EPI) has led to significant decreases in the incidences of vaccine preventable diseases (VPDs) [10]. China’s EPI system makes certain vaccines available at no charge and implements interventions to ensure health protection of children. Interventions include a school-entry vaccination record check program that helps ensure immunization of school-age children, and assuring that migrant children have access to free vaccines regardless of residency [11].

Immunization program successes lead to lower disease rates and decreased visibility of VPDs, but possibly also to complacency among parents about the need to vaccinate [12, 13]. With the decline of vaccine-preventable diseases, rare adverse events following immunization can become more apparent. Combined with inaccurate information about vaccine safety, the relative invisibility of a disease can lead some parents to be hesitant, or even refuse, to vaccinate their child [7, 14]. Vaccine refusal or hesitancy can result in missed or delayed immunization, posing a risk to the child and, for some diseases, to the public. For example, in 2013, the hepatitis B vaccine was widely reported by the media to cause infant deaths in China. An investigation showed the deaths to be coincidental, but the event ultimately resulted in the temporary suspension of a safe vaccine and was associated with a decline of parental confidence and refusal to vaccinate their children [15]. Loss of confidence in vaccines appears to materialize in every province of China, including in less-developed central and western regions and in poor rural areas.

The overall coverage rate of EPI vaccines in China is above 90%, as measured at the township level. However, coverage rates are less than 90% in half of the western province townships. Lack of information about vaccines is a factor associated with missed opportunities to immunize in these areas [16]. Barriers to vaccination need to be addressed in China’s immunization program, particularly in remote and undeveloped areas where the immunization system is weaker [17].

One barrier is financial. To address this, the central government increased public health spending in 2009, and has directed a disproportionately larger share of funding towards the less developed and more rural western and central regions [18–21]. Another barrier is a lack of knowledge about the benefits and risks of vaccination. Communication is a key tool to increase such knowledge. If implemented strategically and integrated into the program, communication can improve public trust and acceptance of vaccination, especially among hard-to-reach populations [14, 22–24].

Communication for Development (C4D) is a systematic, evidence-based, strategic process to promote positive and measurable change at the individual, family, community, social, and policy levels of a society ﻿[25]. Initially developed by the United Nations Children’s Fund (UNICEF), “C4D aims to promote dialogue within communities and with decision-makers at local, national, and regional levels for the purpose of promoting, developing, and implementing policies and programs that drive positive and healthy behavior and social change” [26]. When using the core C4D steps of situation analysis, strategic design, plan development, implementation, and monitoring and evaluation, the C4D strategy has proven to be an effective method to enhance communication skills of immunization professionals to transmit health knowledge, increasing the public’s recognition of the importance of vaccination [27].

In the United States, C4D plays an important role in the promotion of influenza vaccination, and includes messages that take into account knowledge, concerns, and beliefs of targeted populations and of health care professionals [28–31]. Appropriate messages and promotional materials help to enhance the acceptance of vaccination [32].

There is a paucity of information in the literature about communication interventions that measurably raise vaccine coverage [23]. Communication activities are conducted frequently, for example during Immunization Week and when implementing supplementary immunization activities, but these activities are seldom evaluated.

C4D has been used for EPI work in China, but without evaluation. In 2013 and 2014, nine western provinces used C4D to strengthen routine immunization and increase vaccine uptake by focusing on the national goal of eliminating measles. In this study, we evaluated the impact of provincial C4D interventions on caregiver knowledge of immunization services, measles disease, and measles vaccines, and their children’s measles vaccination coverage.

## Methods

### Setting and C4D interventions

The National Health and Family Planning Commission of China, in collaboration with UNICEF, conducted a one-year project called “Communication for Development of Immunization Programs” that began in April 2013. The project focused on measles elimination and was implemented in the Inner Mongolia, Guangxi, Chongqing, Guizhou, Tibet, Shaanxi, Gansu, Ningxia, and Qinghai provinces. These provinces had not previously employed C4D interventions to target the elimination of measles. The strategy’s purposes were to improve immunization professionals’ capacity to disseminate health knowledge, to enhance public awareness and acceptance of vaccination, to help caregivers make informed decisions about vaccinating their children, and to improve coverage levels of EPI vaccines. We selected one county at random from each province and C4D activities were conducted in seven to ten townships selected at random from each of the selected counties.

Based on challenges and characteristics of pilot areas that were identified in a situation analysis conducted with provinces prior to the C4D intervention, provincial Centers for Disease Control and Prevention designed specific plans for C4D activities and organized a series of activities that adhered to local culture. Specific C4D activities were tailored for different regions according to target population characteristics. The following key messages were disseminated in all locations: vaccination can prevent relevant infectious diseases; a vaccination check will be required for entry into kindergarten and primary school; migrant children are eligible for free vaccination; and measles vaccine can stop and prevent measles outbreaks. Specific C4D interventions are summarized in Table 1.

**Table 1:** C4D intervention activities for various target populations in 9 provinces

Target population	Provinces	C4D intervention	Activity description
Migrant children caregivers	Guangxi, Guizhou, Ningxia, Shaanxi	Communication house-to-house at specific time	Household publicity conducted during the spring festival when migrant workers came back home for family reunions.
Caregivers with low education	Gansu, Ningxia, Qinghai, Chongqing	Face-to-face communication and household publicity with inclusion of vaccination messages in items of daily use	Face-to-face communication for caregivers to understand vaccination. Placing messages in items of daily use (calendars, shopping bags) to increase the frequency of caregiver contact with immunization information.
Caregivers with religious belief	Ningxia, Tibet	Social mobilization from religious leaders	Religious leaders to mobilize followers for immunization program during gatherings or public activities.
Ethnic minority caregivers	Inner Mongolia, Tibet	Development of bilingual publicity materials	Creating material about immunization in both Mongolian and Chinese in Inner Mongolia and in both Tibetan and Chinese in Tibet.
Pregnant women	All 9 provinces	Talks and discussions for pregnant women	Conducting immunization talks for pregnant women at Women’s Homes or maternal and child health centers.
Left-behind children caregivers	Gansu, Ningxia, Qinghai, Chongqing	Peer education and face-to-face communication	One caregiver was chosen as a peer educator who learned about the benefits of vaccination through receiving training on immunization and spread vaccination knowledge among other caregivers of the left-behind children.
Caregivers in general	All 9 provinces	Kindergarten entry vaccination check	Publicity activities were carried out in kindergartens, with the help of education departments during the school entry check for vaccination status.

A wide variety of C4D activities were conducted in the nine provinces, which together contained approximately 1.05 million target persons. Fifty-nine training activities were held that had more than 4 300 attendees. C4D activities consisting of personal communications were held 3 000 times, includingcommunication house-to-house, lectures, and classes for pregnant women about vaccination, and peer education. Mass communication methods, including radio broadcast, TV promotion, and display boards and banners, were used more than 800 times. Posters and leaflets were distributed at approximately 1 400 settings, including large events in urban areas and in places of worship. Provinces distributed more than 200 000 leaflets and brochures about vaccination and measles prevention and control, 20 000 environment-friendly shopping bags, and more than 20 000 daily use items such as calendars and towels. Approximately 25 000 pieces of educational material were printed.

### Data collection

We used a before-and-after study design to evaluate the impact of C4D activities. This study design allowed us to focus on changes in knowledge and vaccination coverage, while putting most of the available resources into the interventions. Surveys were conducted in participating townships before and after the C4D interventions. All administrative villages in participating townships were listed and numbered. Three villages from the participating townships were selected using simple random sampling to participate in the survey. In each selected village, after interviewing the first randomly-identified family (regardless of whether there was a survey-age-eligible child in the household), investigators interviewed nearby families until ten caregivers, whose children were born between 1 July 2006 and 30 June 2013, were interviewed using a structured questionnaire.

Baseline and evaluation surveys were conducted face to face. Collected demographic data included caregiver relationship with the children, race/ethnicity, and educational background. Immunization information collected included parental knowledge of measles vaccination, factors influencing caregivers’ decision about vaccinating their children, and caregiver preference for their source of knowledge about vaccines. In the evaluation survey, we asked about caregiver participation in C4D activities as well as caregiver knowledge about the school vaccination check and migrant children’s access to vaccines. The children’s measles vaccination status was obtained from official vaccination certificates.

### Statistical analysis

Answers to seven questions were used to evaluate the caregivers’ vaccination knowledge. These included questions regarding caregiver application for and use of the official provincial vaccination certificate, symptoms of measles, and mode of transmission of measles. Chi-square tests were used to compare knowledge before and after C4D interventions. Statistical analyses were performed with SPSS version 19.0 (International Business Machines Corporation, USA) and Microsoft Excel (Microsoft Company, USA).

Active participation in vaccination was confirmed if caregivers answered “yes” when asked if they were willing to have their children vaccinated. The percentage of caregivers participating in C4D activities and their preferred types of C4D activities were also elucidated.

Whether a child received age-appropriate measles-containing vaccine (MCV) was based on his/her date of birth and date of measles vaccination. Official vaccination cards were used to determine vaccination status. Coverage of MCV was estimated by dividing the number of age-appropriate vaccinated children by the number of children eligible for the measles vaccine.

## Results

### Demographic characteristics of participants

A total of 2 107 households were included in the baseline survey and 2 070 households were included in the evaluation survey. Demographic characteristics of respondents were similar in the baseline and the evaluation surveys.

### Caregiver knowledge of vaccination

Knowledge about vaccination increased by more than 20 percentage points between the baseline and evaluation surveys. After conducting C4D activities, in all provinces, 95% of caregivers were aware of the vaccination record check for entry into kindergarten and primary school, and 80% of caregivers were aware that migrant children were eligible for free vaccination. Overall, more than 70% of caregivers indicated that measles is a respiratory-spread infectious disease, and 90% of caregivers responded correctly to questions about symptoms of measles (see Table 2).

**Table 2:** Caregiver [n (%)] vaccination knowledge during the baseline survey (*N* = 2107) and the final evaluation of (*N* = 2070) the C4D intervention activities

Questions	Baseline survey (%)	Final evaluation (%)	χ^2^	*P* value
Application time for immunization certificate	1 221(57.98)	1 789(86.59)	424.97	<0.0001
Immunization check for kindergarten entry	1 535(72.89)	1 952(94.39)	350.90	<0.0001
Immunization check for primary school entry	1 536(72.93)	1 958(94.68)	361.83	<0.0001
Free vaccination for migrant children	918(43.59)	1 645(79.55)	569.18	<0.0001
Infectiousness of measles	1 290(61.25)	1 835(88.69)	417.35	<0.0001
Measles is a respiratory spread disease	539(25.59)	1 480(71.53)	881.9536	<0.0001
Measles is characterized by fever and rash	1 438(68.28)	1 862(90.04)	298.3715	<0.0001

Caregivers should apply for an immunization certificate for each child within 1 month of his or her birth

### Caregiver willingness to vaccinate their children

**Fig. 1:**
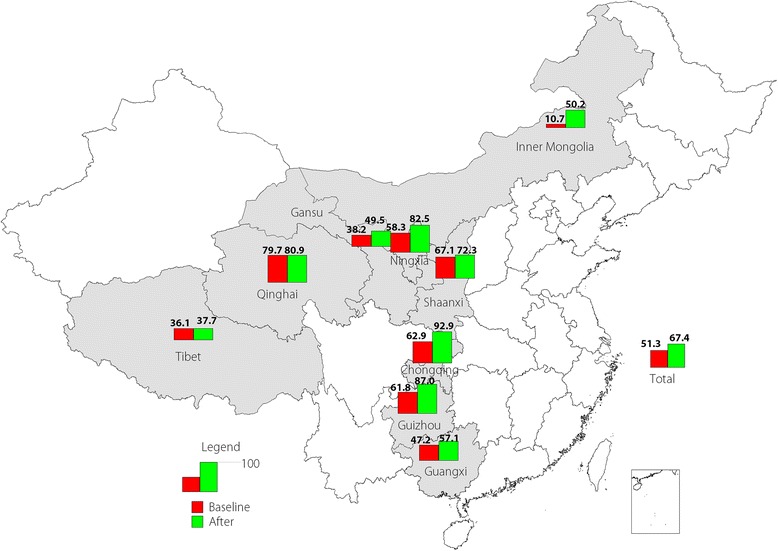
Active vaccination rates by provinces

After conducting C4D activities, caregivers in all provinces were more willing to have their children vaccinated, with the percentage of active vaccination increasing from 51.3% at baseline to 67.4% in the evaluation survey. The rate of active vaccination in Chongqing was over 90% after C4D, but rates of active vaccination in Guangxi, Gansu, Tibet, and Inner Mongolia remained low, at less than 60% (see Fig. 1).

### Vaccination coverage

**Fig. 2:**
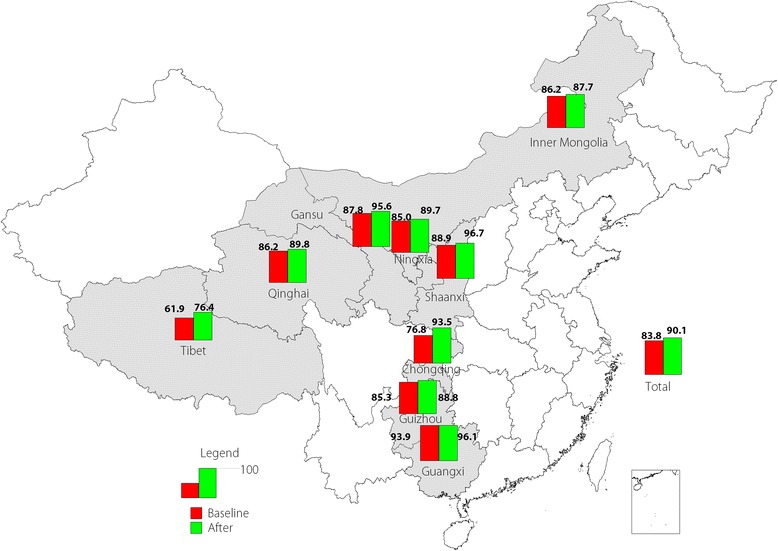
Coverage rates of the first dose of MCV in each studied province

Coverage with at least one dose of MCV was 83.8% at baseline and increased to 90.1% after the implementation of C4D intervention activities. Coverage rates of the first dose of MCV were more than 95% in Guangxi, Shaanxi, and Gansu, but coverage was less than 80% in Tibet (see Fig. 2).

**Fig. 3:**
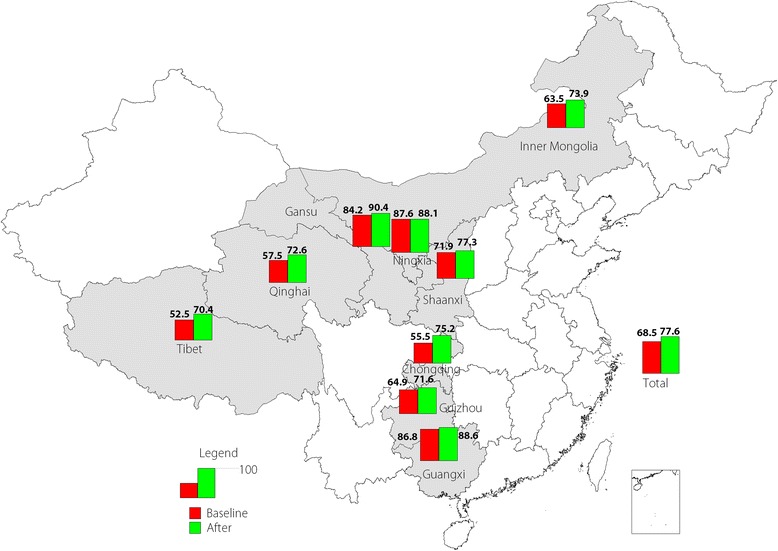
Coverage rates of the second dose of MCV in each studied province

Coverage with two doses of MCV increased from 68.5 to 77.6% between baseline and evaluation. Coverage rates of MCVs were more than 85% in Ningxia, Guangxi, and Gansu. After C4D intervention activities in Guangxi, two-dose MCV coverage was over 90%, but coverage levels in Qinghai, Inner Mongolia, Chongqing, and Tibet were less than 80% after C4D (see Fig. 3).

### Understanding of and participation in C4D activities

**Fig. 4:**
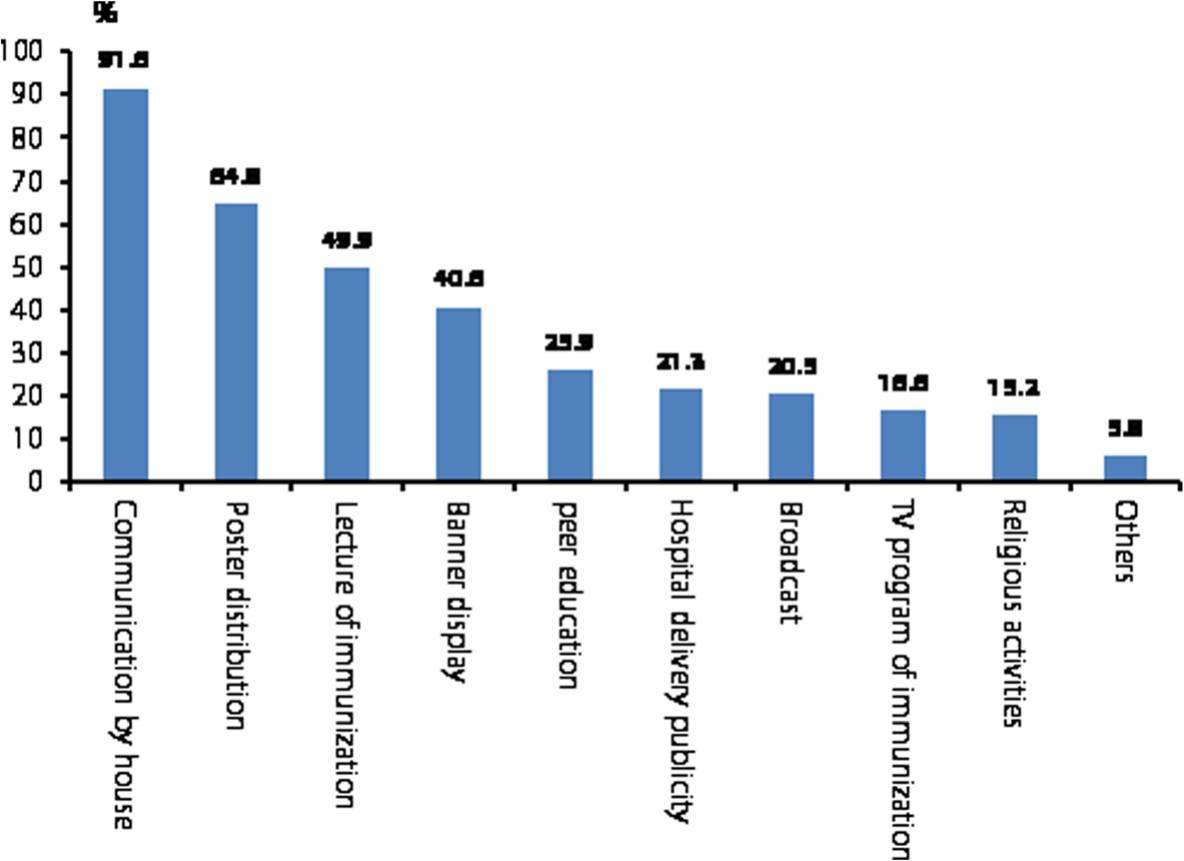
Caregiver participation in C4D intervention activities

The three most popular C4D activities of respondents were house-to-house communications, distribution of leaflets and posters, and lectures on immunization. Acceptance rates for these activities were 91.6, 64.8 and 49.9%, respectively (see Fig. 4).

Among the respondents, 97.5% (1 885/1 933) indicated that participation in C4D activities was helpful to understand components of the immunization program in which they were interested; 82.0% considered that C4D activities could help them understand vaccines better; and 63.8% selected household publicity as their preferred C4D activity.

## Discussion

Based on the before-and-after survey results, C4D intervention activities resulted in increased caregiver knowledge of measles and measles vaccination, increased caregiver awareness of programs that provide access to vaccines for migrant children and ensure vaccination at school entry, and increased receipt of measles vaccines among the caregivers and increased coverage with measles vaccine among children. Increased knowledge, awareness, and coverage may demonstrate a greater appreciation of the importance of vaccination among parents, which could lead to increased immunization coverage in the western provinces of China.

Our evaluation also showed that although coverage increased, vaccination rates for two doses of MCV were still less than 90%, which is below the coverage threshold of 95% for the elimination of measles and maintenance of measles elimination status [33]. Therefore, despite the increase in coverage associated with C4D, we believe that improving immunization coverage to such a high level cannot rely solely on communication activities and that additional interventions, financing increase on routine service, training and supervision of routine immunization program, are required [34–37].

### Study in the context of scientific literature

Acceptance of vaccination is a behavior resulting from a complex decision-making process that can be influenced by many factors [27, 37, 38]. Weaknesses in the immunization delivery system, such as limited availability of vaccination services, a poor clinic environment, and immunization professionals having insufficient knowledge or communication skills may lessen the effectiveness of C4D. A systematic review published in 2011 by Rainey and colleagues showed that approximately 44% of the reasons linked to being under-vaccinated were related to immunization systems, while 28% were linked to parental attitudes and knowledge [39]. This implies that only when the immunization delivery system is adequate will supplementary help of communication activities be sufficient to attain coverage goals.

Most survey respondents indicated that participation in C4D activities fulfilled their interests in the immunization program, and most believed that the relevant authorities should organize more C4D activities. More than half of survey respondents who participated in C4D activities indicated that house-to-house communication was their preferred type of C4D activity. However, this type of communication is time-consuming and costly, and is not feasible to conduct frequently, as scarcity of communication resources limits capacity to support immunization programs [23]. Faced with this challenge, it is well worth considering how a program should most effectively use limited resources to conduct communication activities.

That communication interventions should be tailored to targeted populations or individuals is the essence of implementing a C4D project. Evidence and experience suggest that health-related communications are often more effective when tailored with insights gained from a situation analysis about the targeted populations [40–42]. Listening to the targeted populations (their beliefs, attitudes, and behaviors) and translating this information into communication materials could help achieve desired results. This was the strategy that we undertook to implement our C4D project. We obtained relevant data through a situation analysis and the baseline survey, and we conducted several analyses to develop a sense of the target population’s needs, religions, current behaviors, and culture. Implementation plans and C4D activities were developed for different areas using these analyses. For example, local immunization professionals realized that there were not enough communication materials in the minority language in Tibet, and so the public could not obtain relevant vaccination knowledge in a timely manner. As a response, these professionals developed bilingual Tibetan calendars with key immunization messages displayed at the bottom of the calendar, which proved to be welcomed by Tibetans.

We also worked with religious leaders to disseminate immunization knowledge. Mobilizing populations through religious leaders has proven to be an effective communication tool to promote polio eradication efforts in polio-endemic countries [10, 27]. In our baseline survey, we found that many ethnic minorities had their own beliefs and trusted some religious leaders more than others. In the Ningxia Autonomous Region, people often go to mosques for religious gatherings and they tend to listen to imams very carefully. Therefore, when planning C4D interventions, immunization professionals first showed imams that vaccination is an effective public health method to prevent certain infectious diseases, and then taught them how to disseminate relevant knowledge. The imams used opportunities of religious gatherings to give talks about immunization. This strategy may be more effective than utilizing immunization professionals to disseminate the same content to the local population.

Based on the C4D project experience, targeted communication to improve vaccination uptake should be developed for different groups [27]. Various communication materials should be designed not only for improving the immunization knowledge level of the public, but also for encouraging the public to actively seek vaccination services and get timely vaccinations.

### Limitations

Our study has several limitations that should be considered when interpreting the results. Baseline and evaluation survey respondents were selected at random from the same villages and therefore some respondents may have participated in both surveys. Participation in both surveys could bias the results to show greater impact of C4D activities. Because 1 year transpired between surveys and since the sampled fraction of the population was small, this bias is also likely to be small. Another potential limitation is self-reporting bias, which may have resulted in some caregivers reporting desired responses rather than true responses. However, self-reporting bias would not impact knowledge or coverage, and so this bias is also likely to be small.

As this was a before-and-after study without a concurrent control group, associations may have been influenced by secular trends and results could have been confounded. Immunization service delivery may have improved during the study period, for example, and the study period encompassed the National Children Immunization Day. Vaccination decisions can be influenced by many factors. However, since the study was conducted over a relatively short, one-year period, and since knowledge and coverage are unlikely to change so quickly, we believe that the associations observed were unlikely to be caused by secular trends.

### Recommendations

We believe that the C4D project in western China demonstrated highly desirable results. It appeared to increase caregiver knowledge about vaccination and appeared to encourage caregivers to actively seek immunization services, leading to improved coverage in the target population. The apparent success of the C4D project shows that tailored communication strategies, based on insights gained from situation analyses, can make a significant difference to the behaviors of target populations. We therefore recommend that C4D should be implemented on a larger scale in China, and that selected communication activities should be organized in regions with low vaccination coverage. C4D has the potential to help the public understand the importance of protecting children through vaccination and may be able to increase the demand for immunization services.

## Conclusions

C4D activities helped to increase caregiver immunization knowledge, encouraged caregivers to actively seek immunization services, and improved immunization coverage levels. Tailored communication strategies, based on insights gained in situation analyses, can make a significant difference to the behaviors of targeted populations. The C4D experience should be implemented on a larger scale in China.

## Additional file

Additional file 1: Multilingual abstracts in the five official working languages of the United Nations. (PDF 803 kb)

## Abbreviations

C4D: Communication for development
EPI: Expanded Program on immunization
MCV: Measles-containing vaccine
UNICEF: United Nations Children’s Fund
VPD: Vaccine preventable disease

## References

[1] Bhutta ZA, Sommerfeld J, Lassi ZS, Salam RA, Das JK (2014). Global burden, distribution, and interventions for infectious diseases of poverty. Infect Dis Poverty.

[2] Bonds MH, Keenan DC, Rohani P, Sachs JD (2010). Poverty trap formed by the ecology of infectious diseases. Proc Biol Sci.

[3] Boatin B (2008). The onchocerciasis control progarmme in West Africa (OCP). Ann Trop Med Parasitol.

[4] United Nations. Millennium Development Goals. http://www.un.org/millenniumgoals/.Accessed 17 Feb 2017.

[5] Marsh K (2010). Research priorities for malaria elimination. Lancet.

[6] mal ERACGoM (2011). A research agenda for malaria eradication: modeling. PLoS Med.

[7] Healy CM, Montesinos DP, Middleman AB (2014). Parent and provider perspectives on immunization: are providers overestimating parental concerns?. Vaccine.

[8] Waisbord, S. & Larson, H. (June 2005). Why Invest in Communication for Immunization: Evidence and Lessons Learned. A joint publication of the Health Communication Partnership based at Johns Hopkins Bloomberg School of Public Health/Center for Communication Programs (Baltimore) and the United Nations Children’s Fund (New York).

[9] Yaqub O, Castle-Clarke S, Sevdalis N, Chataway J (2014). Attitudes to vaccination: a critical review. Soc Sci Med.

[10] Commission. CNHaFP (2015). Progress of China’s disease control and prevention.

[11] Guoqiang W (2015). A 60-year history of disease prevention and control in China.

[12] Poland GA, Jacobson RM (2011). The age-old struggle against the antivaccinationists. N Engl J Med.

[13] Smith M (2015). Vaccine safety: medical contraindications, myths, and risk communication. Pediatr Rev.

[14] Atwell JE, Salmon DA (2014). Pertussis resurgence and vaccine uptake: implications for reducing vaccine hesitancy. Pediatrics.

[15] Yu W, Liu D, Zheng J, Liu Y, An Z, Rodewald L (2016). Loss of confidence in vaccines following media reports of infant deaths after hepatitis B vaccination in China. Int J Epidemiol.

[16] Zheng JS, Cao L, Guo SC, et al. Immunization coverage of the national immunization program vaccines at the township level, based on a survey conducted by provincial CDCs in China, 2013[J]. Chin J Vacc Immunizat. 2014;20(6):492–8.

[17] Luo HM, Zhang Y, Wang XQ, Yu WZ, Wen N, Yan DM, et al. Identification and control of a poliomyelitis outbreak in Xinjiang, China. N Engl J Med. 2013;369(21):1981–90. 10.1056/NEJMoa1303368 24256377

[18] Tang S, Ehiri J, Long Q. China’s biggest, most neglected health challenge: non-communicable diseases. Infect Dis Poverty. 2013;2(1):7. 10.1186/2049-9957-2-7 PMC3710110 23849054

[19] Ackumey MM, Gyapong M, Pappoe M, Maclean CK, Weiss MG. Socio-cultural determinants of timely and delayed treatment of buruli ulcer: implications for disease control. Infect Dis Poverty. 2012;1(1):6. 10.1186/2049-9957-1-6 PMC3710079 23849228

[20] Wei X, Zou G, Yin J, Walley J, Yang H, Kliner M, et al. Providing financial incentives to rural-to-urban tuberculosis migrants in Shanghai: an intervention study. Infect Dis Poverty. 2012;1(1):9. 10.1186/2049-9957-1-9 PMC3710084 23849348

[21] Yip WC, Hsiao WC, Chen W, Hu S, Ma J, Maynard A (2012). Early appraisal of China’s huge and complex health-care reforms. Lancet.

[22] So AD, Ruiz-Esparza Q (2012). Technology innovation for infectious diseases in the developing world. Infect Dis Poverty.

[23] Goldstein S, MacDonald NE, Guirguis S, Hesitancy SWGoV (2015). Health communication and vaccine hesitancy. Vaccine.

[24] Mbabazi WB, Tabu CW, Chemirmir C, Kisia J, Ali N, Corkum MG (2015). Innovations in communication technologies for measles supplemental immunization activities: lessons from Kenya measles vaccination campaign, November 2012. Health Policy Plan.

[25] Schiavo R, May Leung M, Brown M (2014). Communicating risk and promoting disease mitigation measures in epidemics and emerging disease settings. Pathog Glob Health.

[26] Fund. UNICEF (2013). Regional communication strategy development guide for newborn care and the prevention and control of childhood pneumonia and diarrhoea in East Asia and the pacific region.

[27] MacDonald NE, Hesitancy SWGoV (2015). Vaccine hesitancy: definition, scope and determinants. Vaccine.

[28] Uscher-Pines L, Maurer J, Kellerman A, Harris KM (2010). Healthy young and middle age adults: what will it take to vaccinate them for influenza?. Vaccine.

[29] Opel DJ, Diekema DS, Lee NR, Marcuse EK (2009). Social marketing as a strategy to increase immunization rates. Arch Pediatr Adolesc Med.

[30] Thompson MG, Gaglani MJ, Naleway A, Ball S, Henkle EM, Sokolow LZ (2012). The expected emotional benefits of influenza vaccination strongly affect pre-season intentions and subsequent vaccination among healthcare personnel. Vaccine.

[31] Opel DJ, Robinson JD, Heritage J, Korfiatis C, Taylor JA, Mangione-Smith R (2012). Characterizing providers’ immunization communication practices during health supervision visits with vaccine-hesitant parents: a pilot study. Vaccine.

[32] Nowak GJ, Sheedy K, Bursey K, Smith TM, Basket M (2015). Promoting influenza vaccination: insights from a qualitative meta-analysis of 14 years of influenza-related communications research by U.S. Centers for disease control and prevention (CDC). Vaccine.

[33] Heywood AE, Gidding HF, Riddell MA, McIntyre PB, MacIntyre CR, Kelly HA (2009). Elimination of endemic measles transmission in Australia. Bull World Health Organ.

[34] Uddin MJ, Saha NC, Islam Z, Khan IA, Shamsuzzaman, Quaiyum MA (2012). Improving low coverage of child immunization in rural hard-to-reach areas of Bangladesh: findings from a project using multiple interventions. Vaccine.

[35] Oyo-Ita A, Nwachukwu CE, Oringanje C, Meremikwu MM (2011). Interventions for improving coverage of child immunization in low- and middle-income countries. Cochrane Database Syst Rev.

[36] Fredrick T, Murhekar MV, Jayaraman Y, Ponniah M, Pattabi K, David JK (2015). Target intervention to increase measles vaccination coverage by identifying low-coverage areas using lot quality assurance sampling, Chennai, India, 2012. Indian J Public Health.

[37] Adams J, Bateman B, Becker F, Cresswell T, Flynn D, McNaughton R (2015). Effectiveness and acceptability of parental financial incentives and quasi-mandatory schemes for increasing uptake of vaccinations in preschool children: systematic review, qualitative study and discrete choice experiment. Health Technol Assess.

[38] McNutt L-A, et al. Affluence as a predictor of vaccine refusal and underimmunization in California private kindergartens. Vaccine. 2015, http://dx.doi.org/10.1016/j.vaccine.2015.11.063. 10.1016/j.vaccine.2015.11.063 26679403

[39] Rainey JJ, Watkins M, Ryman TK, Sandhu P, Bo A, Banerjee K (2011). Reasons related to non-vaccination and under-vaccination of children in low and middle income countries: findings from a systematic review of the published literature, 1999–2009. Vaccine.

[40] Nowak GJ, Gellin BG, MacDonald NE, Butler R, Hesitancy SWGoV (2015). Addressing vaccine hesitancy: the potential value of commercial and social marketing principles and practices. Vaccine.

[41] Garcia LD, Velandia-Gonzalez M, Trumbo SP, Pedreira MC, Bravo-Alcantara P, Danovaro-Holliday MC (2014). Understanding the main barriers to immunization in Colombia to better tailor communication strategies. BMC Public Health.

[42] Gowda C, Schaffer SE, Kopec K, Markel A, Dempsey AF (2013). A pilot study on the effects of individually tailored education for MMR vaccine-hesitant parents on MMR vaccination intention. Hum Vaccin. Immunother.

