# Supplementary material for: Implementing the communication for development strategy to improve knowledge and coverage of measles vaccination in western Chinese immunization programs: a before-and-after evaluation
Source: Infect Dis Poverty. 2017 Apr 24;6:47. doi: 10.1186/s40249-017-0261-y (PMC5402053; doi:10.1186/s40249-017-0261-y)
Supplement: Additional file 1: — Multilingual abstracts in the five official working languages of the United Nations. (PDF 803 kb) [file 40249_2017_261_MOESM1_ESM.pdf]

Translation of the abstract into the five official working languages of the United Nations

تنفيذ استراتيجية الاتصالات من أجل التنمية لزيادة الوعي بالتطعيم ضد الحصبة وتحسين نطاق تغطية برامج التطعيم في غرب الصين له: تقييم لما قبل وما بعد تنفيذ الاستراتيجية

مينغ لو، ياو-جو تشو، وين-جو يو، روبرت شربير، يو-كينغ جو، زو جو، كي-رو سو، منغ-غوان دوان، شوان جانغ، فو-كيانغ تسواي، هوا-كينغ وانغ، يي-بياو جو، كينغ-وو جيانغ

الملخص:

الخلفية: استراتيجية الاتصالات من أجل التنمية (C4D) هي استراتيجية تروج لها منظمة الأمم المتحدة للطفولة. تهدف هذه الاستراتيجية إلى تبني تغييرات إيجابية محسوسة على مستوى الفرد والأسرة والمجتمع، وكذلك على المستوى الاجتماعي ومستوى سياسة المجتمع. سبق إجراء أنشطة استراتيجية الاتصالات من أجل التنمية في غرب الصين باعتبارها جزءًا من برامج التطعيم على مستوى المقاطعات. في هذه الدراسة، أجرينا تقييمًا لمدى ارتباط هذه الاستراتيجية بالتغييرات الطارئة في مدى وعي الآباء بخدمات التطعيم وأمراض الحصبة والتطعيم ضدها، وبالتغييرات الواقعة في نطاق تغطية التطعيم ضد الحصبة لأطفالهم.

المنهج: بدءًا من أبريل/نيسان عام 2013 وحتى أبريل/نيسان عام 2014، تم تنفيذ أنشطة استراتيجية الاتصالات من أجل التنمية باعتبارها جزءًا من برامج التطعيم على مستوى المقاطعات في المقاطعات الآتية: منغوليا الداخلية، قوانغشي، تشونغتشينغ، قويتشو، التبت، شنشي، قانسو، نينغشيا، تشينغهاي. قمنا بدراسة من النوع المعتمد على ما قبل وما بعد تنفيذ الاستراتيجية، واستعنّا بالمقابلات التي أجريناها وجهًا لوجه لتقييم التغييرات الناتجة عن الاستراتيجية في مدى وعي الآباء وفي نطاق تغطية برامج التطعيم.

النتائج: أجرينا مسحًا لـ2107 أسرة عند بدأ الدراسة، و2070 أسرة بعد مرور عام واحد على أنشطة استراتيجية الاتصالات من أجل التنمية. بعد تنفيذ الاستراتيجية، أصبح 95% من مقدمي الرعاية على دراية بشرط فحص سجل التطعيمات المطلوب للالتحاق برياض الأطفال والمدارس الابتدائية؛ وأصبح 80% من مقدمي الرعاية على دراية بكون أطفال المهاجرين مؤهلين لتلقي التطعيم مجانًا؛ وأصبح أكثر من 70% من مقدمي الرعاية على دراية بأن مرض الحصبة عبارة عن عدوى بالجهاز التنفسي؛ كما أصبح 90% من مقدمي الرعاية على دراية بأعراض مرض الحصبة. كذلك، زادت رغبة مقدمي الرعاية باصطحاب أطفالهم للعيادات لتلقي التطعيم من 51.3% عند بدأ الدراسة إلى 67.4% في المسح الذي أجري بعد الانتهاء من تنفيذ الاستراتيجية. كما زاد نطاق تغطية التطعيم الذي يحتوي على فيروس الحصبة المكون من جرعة واحدة (MCV) من 83.8% عند بدأ الدراسة إلى 90.1% بعد تنفيذ الاستراتيجية، وزاد نطاق تغطية التطعيم الذي يحتوي على فيروس الحصبة المكون من جرعة واحدة عن 95% في مقاطعات قوانغشي وشنشي وقانسو، في حين زاد نطاق تغطية التطعيم الذي يحتوي على فيروس الحصبة المكون من جرعتين من 68.5% إلى 77.6%. كان الاتصال مع كل أسرة على حدة هو النشاط الأكثر شيوعًا وسط مقدمي الرعاية من بين أنشطة استراتيجية الاتصالات من أجل التنمية (فضّله 91.6% منهم)، يليه الملصقات والمحاضرات التثقيفية (فضّلهما 64.8% و49.9%).

الاستنتاجات: لاستراتيجية الاتصالات من أجل التنمية علاقة بزيادة وعي مقدمي الرعاية بالحصبة، وبزيادة رغبتهم في تلقي أطفالهم لخدمات التطعيم، وبزيادة نطاق تغطية التطعيم ضد الحصبة. قد تتمكن استراتيجيات الاتصالات المصممة على أساس المعرفة المكتسبة من هذه التحليلات من زيادة نطاق تغطية التطعيم للمناطق التي يتعذر الوصول إليها. ينبغي النظر في تنفيذ استراتيجية الاتصالات من أجل التنمية على نطاق أوسع في الصين.

Translated from English version into Arabic by Heba Kandel, through

TRANSLATORS WITHOUT BORDERS

实施交流促发展策略对提高中国西部地区麻疹疫苗接种率和预防接种知识知晓率的影响观察：前后对照研究

陆明、褚尧竹、余文周、Robert Scherpbier、周玉清、朱徐、苏琪茹、段梦娟、张旋、崔富强、王华庆、周艺彪、姜庆五

摘要

目的：联合国儿童基金会倡导的交流促发展策略可促进个体、家庭、社区、社会以及社会政策发生积极的、可测量的改变。在中国西部部分省份免疫规划工作中，实施了交流促发展策略。在这项研究中，我们评估了实施交流促发展策略对改善儿童父母预防接种服务知识知晓情况、麻疹发病和麻疹疫苗使用情况、以及与儿童麻疹疫苗接种率的关联情况等。

方法：2013 年 4 月到 2014 年 4 月间，内蒙古、广西、重庆、贵州、西藏、陕西、甘肃、宁夏和青海省（区、市）在省级免疫规划工作中实施了交流促发展策略。我们通过干预前后对照研究（前后两次横断面调查），采用面对面访谈形式，评估适龄儿童父母相关预防接种知晓情况及疫苗接种率变化情况。

结果：基线调查了 2 107 户家庭，并在采取交流促发展策略干预一年后，随访到 2 070 户家庭。结果显示，实施交流促发展策略后，95%儿童监护人知道入托、入学有查验疫苗接种记录的要求；80%儿童监护人知道流动儿童享有免费接种的权利；超过 70%儿童监护人知道麻疹是一种呼吸道传染病；90%儿童监护人知道麻疹的相关症状。儿童监护人希望带着孩子到接种点接种疫苗的比例由基线调查时的 51.3%提高到干预后的 67.4%。含一剂次麻疹成分疫苗免疫接种率（MCV）从基线调查时的 83.8%上升至 90.1%。广西、陕西和甘肃三省一剂次麻疹疫苗免疫接种率超过 95%，两剂次麻疹疫苗免疫接种率从 68.5%提高到 77.6%。儿童监护人认为，上门宣传是交流促发展策略最有效的手段（喜爱程度达 91.6%），其次是海报宣传和教育讲座（喜爱程度分别为 64.8%和 49.9%）。

结论：交流促发展策略有助于儿童监护人了解麻疹防治的相关知识，有助于提高他们接受儿童免疫服务的意愿，有助于提高麻疹疫苗接种率。基于上述研究制定精细化的沟通策略，可提高边远地区、免疫服务难以企及地区儿童疫苗接种率。交流促发展策略应在中国更多地区推广实施。

Translated from English version into Chinese by Ming Lu

Mise en œuvre d'une stratégie de communication pour le développement visant à améliorer les connaissances et le taux de vaccination anti-rougeole dans le cadre de programmes de vaccination de Chine occidentale: une évaluation préalable et a posteriori

Ming Lu, Yao-Zhu Chu, Wen-Zhou Yu, Robert Scherpbier, Yu-Qing Zhou, Xu Zhu, Qi-Ru Su, Meng-Juan Duan, Xuan Zhang, Fu-Qiang Cui, Hua-Qing Wang, Yi-Biao Zhou, Qing-Wu Jiang

RÉSUMÉ

Contexte: la communication pour le développement (C4D) est une stratégie mise en œuvre par le Fonds des Nations Unies pour l'enfance afin de favoriser des changements positifs et mesurables aux échelons familial, communautaire, social et politique de la société. En Chine occidentale, des activités de C4D ont au préalable eu lieu dans le cadre de programmes de vaccination au niveau provincial. Dans le cadre de cette étude, nous avons évalué l'association de la C4D à des changements des

connaissances parentales sur les services de vaccination, la rougeole, le vaccin anti-rougeole ainsi qu'à des changements en termes de taux de couverture du vaccin anti-rougeole de leurs enfants.

Méthodes: des activités de C4D ont eu lieu d'avril 2013 à avril 2014 dans le cadre de programmes de vaccination provinciaux en Mongolie intérieure ainsi que dans les provinces de Guangxi, Chongqing, Guizhou, Tibet, Shaanxi, Gansu, Ningxia et Qinghai. Nous avons adopté un plan d'étude avant-après et nous avons eu recours à des entretiens en face à face pour évaluer les changements en termes de connaissances des parents et de taux de vaccination.

Résultats: nous avons analysé 2 107 ménages au début et 2 070 ménages après un an d'activités de C4D. À la suite de la C4D, 95 % des aidants avaient été sensibilisés à la nécessité du contrôle du dossier de vaccination préalable à l'entrée au jardin d'enfants et à l'école primaire. 80 % des aidants savaient que les enfants immigrés avaient droit à une vaccination gratuite. Plus de 70 % des aidants avaient été sensibilisés au fait que la rougeole est une maladie infectieuse respiratoire et 90 % des aidants connaissaient les symptômes de la rougeole. La disposition des aidants à emmener leurs enfants à l'établissement de soins de santé en vue de leur vaccination est passée de 51,3 % au début de l'activité à 67,4 % dans le cadre de l'étude post-C4D. Le taux de couverture du vaccin contenant le virus de la rougeole (VCR) monodose a augmenté de 83,8 % au début à 90,1 % après la mise en œuvre de la C4D. Le taux de couverture du VCR monodose était supérieur à 95 % dans les provinces de Guangxi, Shaanxi et Gansu. Le taux de couverture du VCR bi-dose a augmenté de 68,5 % à 77,6 %. La communication à domicile représentait l'activité de C4D la plus populaire parmi les aidants (91,6 %) suivi des affiches et des entretiens de sensibilisation (64,8 % et 49,9 %).

Conclusions: la C4D est associée à un approfondissement des connaissances des aidants à propos de la rougeole, à une hausse de la disposition à rechercher des services de vaccination pour les enfants et à une augmentation du taux de vaccination anti-rougeole. Des stratégies de communication ciblées reposant sur les informations recueillies dans le cadre de ces analyses sont susceptibles d'améliorer le taux de vaccination dans les zones difficiles d'accès. Il convient d'envisager la mise en œuvre de la C4D à grande échelle en Chine.

Translated from English version into French by eric ragu, through

TRANSLATORS WITHOUT BORDERS

Внедрение стратегии «Коммуникация в целях развития» для повышения знаний и обеспечения вакцинации от кори в составе программ иммунизации в Западном Китае: оценка до и после мероприятий

Мин Лу (Ming Lu), Яо Чжу-Чу (Yao-Zhu Chu), Вэнь-Чжоу Юй (Wen-Zhou Yu), Роберт Шерпбиер (Robert Scherpbier), Юй-Цин Чжоу (Yu-Qing Zhou), Сюй Чжу (Xu Zhu), Ци-Жу Су (Qi-Ru Su), Мэн-Цзюань Дуань (Meng-Juan Duan), Сюань Чжан (Xuan Zhang), Фу-Цян Цуй (Fu-Qiang Cui), Хуа-Цин Ван (Hua-Qing Wang), И-Бяо Чжоу (Yi-Biao Zhou), Цин-У Цзян (Qing-Wu Jiang)

АННОТАЦИЯ

Краткое описание. «Коммуникация в целях развития» (КЦР)/(Communication for Development, C4D) – это стратегия, пропагандируемая Детским фондом ООН и стимулирующая позитивные и ощутимые сдвиги на разных уровнях общества, а именно: индивидуальном, семейном, общественном, социальном и законодательном. В Западном Китае мероприятия КЦР ранее включались в программы иммунизации провинций. В данном исследовании мы выполнили оценку влияния КЦР на изменения в знаниях родителей о службах вакцинации, кори и противокоревой вакцине, и о нововведениях в обеспечении их детей этой вакциной.
Методы. С апреля 2013 года по апрель 2014 года мероприятия КЦР входили в состав программ иммунизации провинций Внутренняя Монголия, Гуанси, Чунцин, Гуйчжоу, Тибет, Шэньси, Ганьсу, Нинся и Цинхай. Мы применили метод исследования «до и после» и проводили личные беседы для оценки изменений в знаниях родителей и обеспечении вакциной.
Результаты. Мы опросили 2 107 семейств в начале исследования и 2 070 семейств через год после мероприятий КЦР. После КЦР 95% лиц, ухаживающих за детьми, знали, что для приёма в детский сад и начальную школу необходимо иметь запись о сделанной прививке; 80% знали, что детям иммигрантов прививка делается бесплатно; свыше 70% знали, что корь – это заразное респираторное заболевание, и 90% были знакомы с симптомами кори. Готовность привести своих детей в клинику для вакцинации выросла с 51,3% в начале исследования до 67,4% при опросе после КЦР. Количество прививок одной дозы противокоревой вакцины MCV увеличилось с 83,8% в начале исследования до 90,1% после КЦР. В провинциях Гуанси, Шэньси и Ганьсу обеспечение одной дозой MCV превысило 95%. Количество прививок двух доз MCV выросло с 68,5% до 77,6%. Наиболее популярным способом коммуникации при проведении КЦР был признан обход домов (одобрили 91,6% опрошенных), за которым следуют плакаты и учебно-просветительные лекции (одобрили 64,8% и 49,9% опрошенных).
Выводы. Стратегия КЦР помогла лицам, ухаживающим за детьми, узнать больше о кори, укрепила их готовность прививать своих детей и способствовала росту количества сделанных прививок от кори. Методы коммуникации, приспособленные к местным условиям и основанные на информации, собранной в результате исследования, могут улучшить вакцинацию в труднодоступных районах. КЦР следует внедрять в Китае в более широком масштабе.

Translated from English version into Russian by Natalia Potashnik, through

TRANSLATORS
WITHOUT BORDERS

Aplicación de la estrategia Comunicación para el Desarrollo con el objeto de mejorar el conocimiento y la cobertura de la vacuna contra el sarampión en los programas de inmunización de China occidental: valoración de la situación anterior y posterior a la estrategia

Ming Lu, Yao-Zhu Chu, Wen-Zhou Yu, Robert Scherpbier, Yu-Qing Zhou, Xu Zhu, Qi-Ru Su, Meng-Juan Duan, Xuan Zhang, Fu-Qiang Cui, Hua-Qing Wang, Yi-Biao Zhou y Qing-Wu Jiang

RESUMEN

Antecedentes: Comunicación para el Desarrollo (C4D) es una estrategia promovida por el Fondo de las Naciones Unidas para la Infancia orientada a fomentar cambios positivos y medibles a escala individual, familiar, comunitaria, social, así como en los niveles políticos de la sociedad. En China occidental, las actividades de la estrategia C4D se han llevado a cabo anteriormente como parte de los programas de inmunización a nivel de provincias. En este estudio hemos evaluado la relación que existe entre la estrategia C4D y la mejora de los conocimientos que poseen los padres acerca de los servicios de inmunización, la enfermedad del sarampión y la vacuna contra el sarampión, y los cambios que se observaron en la tasa de vacunación contra el sarampión de sus hijos.
Métodos: en el período comprendido entre abril de 2013 y abril de 2014, se llevaron a cabo las actividades de la C4D en el interior de Mongolia y en las provincias de Guangxi, Chongqing, Guizhou, Tíbet, Shaanxi, Gansu, Ningxia y Qinghai, como parte de los programas de inmunización provincial. Hemos utilizado un diseño de estudio para evaluar la situación anterior y posterior a la aplicación de la estrategia, y hemos realizado entrevistas en persona para evaluar los cambios en lo que respecta a los conocimientos de los padres acerca de la enfermedad y la cobertura de la vacunación.
Resultados: en un principio se encuestaron 2 107 hogares, y tras un año de llevar a cabo las actividades de la estrategia se encuestaron unos 2 070 hogares. Al cabo de la aplicación de la C4D, el 95% de las personas encargadas del cuidado de los niños estaba al tanto de la necesidad de presentar el registro de vacunación como requisito para inscribir a los niños en el jardín de infantes o la escuela primaria; el 80% de esas personas era consciente de que los niños migrantes podían recibir vacunación gratuita; más del 70% de los cuidadores sabían que el sarampión es una enfermedad infecciosa respiratoria, y el 90% de ellos conocían los síntomas de la enfermedad. La disposición de los cuidadores de llevar a sus hijos a las clínicas de vacunación aumentó del 51,3% al 67,4% en el período posterior a la encuesta de la C4D. La cobertura de una dosis de la vacuna contra el sarampión aumentó del 83,8% al 90,1%, antes y después de la C4D, respectivamente. En las provincias de Guangxi, Shaanxi y Gansu, la cobertura de una dosis de la vacuna contra el sarampión superó el 95%. La cobertura de una segunda dosis de la vacuna contra el sarampión pasó del 68,5% al 77,6%. La actividad más practicada de la estrategia C4D fue la propagación oral de información entre las personas encargadas del cuidado de los niños (91,6% a favor), seguida de la difusión de información por medio de afiches y charlas educativas (64,8% y 49,9% a favor).
Conclusiones: la estrategia C4D se asocia con un mayor conocimiento de las personas a cargo del cuidado de niños acerca de la enfermedad del sarampión, una mayor disposición de solicitar servicios de inmunización para sus hijos y una mayor cobertura de la vacunación contra el sarampión. El diseño de estrategias de comunicación específicas basadas en los planteamientos obtenidos de esos análisis podría contribuir a una mayor cobertura de vacunación en las zonas de difícil acceso. La estrategia C4D debería tenerse en cuenta para una implementación a mayor escala en China.

Translated from English version into Spanish by MKaszczynice, through

TRANSLATORS
WITHOUT BORDERS
